# Patients’ preference approach to overcome the moral implications of family-centred decisions in Saudi medical settings

**DOI:** 10.1186/s12910-022-00868-8

**Published:** 2022-12-06

**Authors:** Manal Z. Alfahmi

**Affiliations:** King Abdullah Medical City, Makkah, Saudi Arabia

**Keywords:** Autonomy, Family dominance, Shared decision-making, The preference approach to patient care

## Abstract

**Background:**

In Saudi clinical settings, cultural influences can give a patient’s family authority to override the patient’s autonomous right to make informed health-related decisions. Cultural values should not prevent patients from exercising their genuine preferences when making medical decisions in their own best interests.

**Discussion:**

This article discusses the moral implications of family-centred medical decisions for autonomous patients who are competent and capable of making decisions. The author argues that socio-cultural values do not justify the decision to override patient autonomy when patients express a preference for making their own choices.

**Conclusion:**

The author recommends the use of a model of shared decision-making that accounts for both individual and relational conceptions of autonomy, approaching patients’ preferences in all medical encounters with the aim of minimising the potential for socio-cultural values to undermine patient autonomy. Although this approach is a safeguard against both family and medical paternalism, allowance is made for clinicians to act in weakly paternalistic ways when patients at high risk of exacerbating existing medical conditions are likely to benefit from delaying or limiting the disclosure of potentially distressing but non-fatal diagnoses and prognoses. Thus, the author argues that even in a culture that supports family involvement in management decisions, physicians should respect patient autonomy by asking patients for their preferences in the disclosure of their medical diagnoses, prognoses and management options and verifying patients’ preferences about the roles they wish their families to play (if any) in health-related decisions.

## Background

In 2019, the Saudi Ministry of Health (MOH) [1] published guidelines that cover different aspects of informed consent in Saudi medical and research settings. These guidelines for informed consent [1] stipulate that it is not permissible to override a pregnant patient’s capacity to make decisions and she retains the right to authorise her father, brother, husband, other male or female relatives or even someone outside of her family to give consent on her behalf. However, the following footnote from the same section of the guidelines contradict this messaging, and it is not clear if they intended to be as an example of what should not occur:

Example (4): A 9-month pregnant lady came with embryonic fluid leakage; all vital signs of the embryo were stable permitting normal delivery. After examination, the gynecologist suggests to the husband doing a cesarean section giving that it is easier than normal delivery. The wife refused but the medical team tried again by telling the husband of possible harm to the baby and leading him to force his wife to do the unnecessary cesarean section even without taking the approval of the mother. [1, p. 15].

This footnote does not explicitly state whether the pregnant woman in the example retains or has lost decisional capacity. If she retains decisional capacity, why is the medical team seeking approval for a cesarean section from her husband? If she has lost decisional capacity, why does it state that “the wife refused”? Further, the footnote does not state whether the husband is her representative in decision-making by default or if she nominated him.

The third item under the heading “Special Cases of Informed Consent” from the guidelines [1, p. 14–15] discusses a pregnant patient’s informed consent. This item shows a clear lack of clarity as it does not mention the reasons or under what conditions the pregnant woman might have to delegate her decision-making authority to her husband. If a pregnant woman has full capacity, is she nominating the person who will give consent on her behalf only if she subsequently loses decisional capacity? And if she nominates another person to provide consent on her behalf in case she loses decisional capacity, is that person obligated to make a decision consistent with what she indicated she wanted? Finally, it is not specified what is meant by “as long as the authorization was approved,” as it does not state who would approve the nomination [1, p. 15].

This article examines the ethical implications of giving priority to family-centred decisions over patient-centred decisions in circumstances where those perspectives diverge in medical settings.

The author discusses the extent to which patient autonomy is overridden in Saudi healthcare due to family dominance over patients’ health-related decisions, highlighting the lack of national policies directed specifically against family dominance to acknowledge the implications of this practice for patient care. This is followed by a discussion of the importance of adopting a model of shared decision-making that accounts for both relational and individual conceptions of autonomy.

The adoption of the model of shared decision-making to guide physicians in checking patients’ preferences for medical decision-making in Saudi healthcare can protect patients’ preferences from being overridden by both family paternalism and medical paternalism. The patient preference approach suggested in this article uses the model of shared decision-making, which seeks to protect patients’ autonomous rights to make health-related decisions while respecting cultural and religious influences on patients’ autonomy. Such an approach recommends the practice of checking patients’ preferences in health-related decisions in a more detailed and autonomy-focussed way than that which is currently accepted in Saudi medical settings, where patient consent is obtained but preferences for disclosures and the extent of family involvement in decision-making are inadequately checked. This article aims to reflect on the importance of continuously assessing patients’ preferences for information disclosure and consent, especially during serious medical encounters, as their preferences can change with time and the seriousness of their medical circumstances. Moreover, this article emphasises that cultural values must be respected as long as they do not prevent patients from exercising their genuine preferences when making medical decisions. Thus, the author argues that even in a culture that supports family involvement in management decisions, physicians should respect patient autonomy by asking patients for their preferences in the disclosure of their medical diagnoses, prognoses and management options.

## Patient autonomy in Saudi healthcare

This section identifies a range of current practices in Saudi medical and research settings that give greater weight to the family’s role in medical decisions than to patient autonomy. These practices result in a range of harms, such as a lack of opportunity for patients to consent to medical interventions and research participation for themselves and violations of patient privacy and confidentiality.

Relying on families rather than the patients themselves to make health-related decisions can potentially subvert the ethical principle of respect for patient autonomy and the practice of informed consent. Due to cultural reasons, a competent patient’s family may prevent healthcare practitioners from asking the patient for their preferences for a full or partial disclosure of their diagnosis and prognosis. This practice is harmful when an uninformed patient who is competent enough to make management decisions is prevented from being involved in important health decisions. As Beauchamp and Childress [2, p. 104] suggest, “To respect autonomous agents is to acknowledge their right to hold views, to make choices, and to take actions based on their personal values and beliefs.” An autonomous individual is someone who is self-determining or self-governing, meaning someone with the capacity to act freely on the basis of self-chosen plans, “analogous to the way an autonomous government manages its territories and sets its policies” [2, p. 99].

Thus, respect for patient autonomy in medical settings implies that the patient should be aware of all the facts concerning their condition and not prevented from making decisions for their best interest [3]. When the informed patient consents for a health-related decision, their consent has to be voluntary without any controlling influences by others, including their family, as the family’s values and priorities might differ from those of the patient and might result in the family making decisions not in the patient’s best interest. However, Ho [3, p. 129] argues that “family involvement and considerations of family interests can be integral in promoting patients’ overall agency.” This means that family involvement can be essential in promoting patients’ interests when the patient prefers their family to be involved in the patient’s healthcare decisions.

In the late 1970s, Beauchamp and Childress [2, p. 3] introduced the four principle approach to biomedical ethics, which is also known as principlism. This approach includes the principles of respect for patient autonomy, non-maleficence, beneficence and justice. Beauchamp and Childress [2] claim that the four principles approach is drawn from the “common morality,” which they describe as “the set of universal norms shared by all persons committed to morality.” Thus, the four principles approach considers the obligations, rules and virtues that healthcare professionals in the medical field must follow. The approach incorporates forms of ethical reasoning drawn from deontological and consequentialist frameworks as well as virtue ethics to define professional obligations that seek to maintain patients’ rights and promote the best outcomes in terms of the patients’ interests. It is intended to apply to all healthcare professionals in all medical settings regardless of cultural and religious backgrounds [2].

In Western bioethics, the emphasis given to the principle of respect for autonomy reflects liberal political values that prioritise individual interests over familial and community interests. This is based on the liberal presumption that individuals are best positioned to know what is in their own best interests [4]. Thus, even if patients choose to follow certain authorities, such as cultural traditions or religious beliefs, they can still be autonomous in making decisions as long as their decisions are based on their personal preferences, values and beliefs and there is no deception, coercion or manipulation by others. In accordance with Mill’s harm principle, the principle of respect for patient autonomy supports autonomous decisions and actions as long as these decisions do not harm or restrict the autonomy of others [4].

Kazdaglis et al. [5] note that healthcare practitioners may make general assumptions about their patients’ preferences based on their previous experiences with patients from the same culture. Further, Kazdaglis et al. [5] emphasise that professionals should ask every patient for their preferences for diagnosis disclosures and not depend solely on their cultural values. For example, the norm of full disclosure in a particular culture does not indicate that physicians should practise full disclosure with all patients since every patient has unique preferences, feelings and thresholds for coping with the facts about their health [5].

According to Aljubran [6, p. 142], the “complicated political, social, and religious mix of values” that form the socio-cultural context of Saudi Arabia can influence public attitudes to be more conservative towards full disclosure in medical settings. Alahmad and Silverman [7, p. 199] explain these values by noting that “Saudi Arabia is ruled by a royal family and the king is the head of the council of ministers, which issues the laws,” and that “these laws must not contradict Islam,” noting that Saudi Arabia has “a tribal social structure with strong extended family relationships.” This conservative community dynamic often enhances the protective power of the family over individual members who become patients. Thus, Aljubran [6] emphasises that healthcare practitioners must understand the effects of these values in addition to their psychosocial and educational influences when responding to public attitudes that harbour less respect for patient autonomy in deference to the role of the family.

Kazdaglis et al. [5] examine patients’ attitude towards full disclosure in the United Kingdom (UK), finding that 13% of patients prefer to only be informed about good news. This suggests that not all patients prefer the complete truth and that due to an overestimation of individual autonomy and individualism in Western countries, physicians might disclose too much information without first asking patients for their preferences.

In Saudi Arabia, while physicians generally acknowledge the capacity for competent patients to govern their own health, it is common for physicians to initially disclose a patient’s diagnosis and prognosis to their family or to attempt to conceal that information from the patient at the request of their family, regardless of the condition the patient has. This is widely accepted and practised because it is believed to prevent a patient from experiencing additional physical and psychological stress [8]. Sharing a patient’s private medical information with family members without the patient’s authorisation is a clear breach of confidentiality, as confidentiality involves protecting patients’ private information so that they maintain trust within the doctor–patient relationship (among other reasons) [2].^1^ Just as physicians expect their patients to honestly disclose medically relevant information and sensitive issues, patients expect their physicians to honestly disclose relevant information, such as the severity of their diagnoses, prognoses, treatment options and the side effects of the therapies proposed. Physicians are also expected to keep their patient’s medical information confidential unless the patient authorises disclosure or, as in the case of contagious diseases, the diagnosis requires disclosure. Thus, mutual trust and respect are intrinsically valuable to doctor–patient relationships. Central to fostering these values is the principle of respect for patient autonomy, which should be universally applied regardless of socio-cultural influences.

According to Aljubran [6], physicians in Saudi Arabia may avoid truthfully disclosing their patients’ diagnoses and prognoses because they often feel overwhelmed by the prospect of breaking bad news. They may also fear their patients’ reactions to bad news or believe that this process will take up too much time in their busy schedules. Therefore, they may prefer to avoid confronting or harming their patients by disclosing such information to their families instead. Furthermore, Bou Khalil’s [8] review of attitudes, beliefs and perceptions about the truthful disclosure of cancer-related information in the Middle East found that there is a lack of official policies in Saudi Arabia that are designed to protect patients’ rights to privacy and confidentiality in relation to their medical information.

Kazdaglis et al. [5] note that healthcare professionals have a duty to professionally deliver bad news to patients after considering the sensitivity of the information and its impact on the patient’s quality of life, including their emotional, social and physical wellbeing and their ability to perform daily tasks. Handling such information in an insensitive way can be as detrimental as hiding it from the patient. That healthcare practitioners may lack the professional skills needed to break bad news provides some, albeit only weak, support to a family’s demands for non-disclosure, so that they may spare the patient from the harmful impacts of a physician’s deficient communication skills and empathy. Based on these considerations, Kazdaglis et al. [5] conclude that healthcare professionals must tell the truth when patients clearly express the desire to know it, but avoid telling the whole truth when patients might not want to hear it. They also posited that while telling the full truth to a patient is preferable from a philosophical point of view, it may not always be the best course of action in real life.

Physicians who withhold health information from patients or lie to them due to socio-cultural influences, amongst other reasons, fail to meet their moral obligations if they allow families to dominate patients’ health-related decisions without legitimate justification. However, as valuable as these obligations are, it is sometimes morally justifiable to delay the disclosure of a bad diagnosis when there are significant concerns related to patient safety and welfare; this is known as “physicians’ therapeutic privilege” [2, p. 126]. Therapeutic privilege remains highly controversial from an ethical perspective. For therapeutic privilege to be justified, it must be motivated by medical rather than socio-cultural reasons.

Practices involving male-relative guardianship in Saudi Arabian society have likely contributed to weaker protections of patient autonomy, especially amongst women. Although the notion of guardianship predominately relates to protecting and supporting vulnerable individuals, such as those who are younger or incompetent, in Saudi Arabia this protection extends to wives, daughters and even elderly family members. Thus, the autonomy of female and elderly patients is often overridden by male relatives during health-related decisions [9].

Muaygil [10] discusses the impact of training physicians in cultural competence, which has been found to undermine Saudi female patients’ autonomy when making treatment decisions in Western medical settings. Muaygil [10] examines a case in an American hospital where a Saudi husband overrode his wife’s autonomy to consent to a surgical operation, concealing its possible complications to her fertility. Muaygil [10] criticises the attending physician—who was trained to accept cultural differences—for allowing such practices under the moral justification of respecting the norms of other cultures. She further claims that training physicians to prioritise cultural competence in relation to patient care may be more harmful than beneficial if it leads physicians to apply cultural generalisations to all patients from a particular culture without asking for their personal preferences for medical decisions.

Many believe that Saudi women accept the dominant role of their male relatives in making medical decisions that are in their best interest [6]. However, Muaygil [10] rejects the notion that Saudi women prefer to be dependent on their male relatives or that this dependency is more highly valued than autonomy. Muaygil’s argument is made against the background of Saudi women’s increased access to education and more financial independence through improved employment opportunities in the country. That women do not value the domination of their male relatives is also evident from the emergence of the Saudi women’s movement, which has criticised certain regulations and laws, such as those preventing women from driving, and called for the elimination of the guardianship system.

In Altoaimy’s [11] article about changes to laws that now allow women to drive in Saudi Arabia, she analysed Twitter debates before the ban on female driving was lifted, finding that social media use, among other contributing factors, is driving a movement of cultural change.^2^ She also notes that increasing demands to improve women’s rights made on Twitter, along with other contemporary pressures, have likely prompted the Saudi government to change many of their policies for women; for example, enabling women over the age of 21 to freely travel abroad and to obtain passports without the permission of their male guardians [11].^3^ Such changes have supported a shift towards greater independence for women in Saudi Arabia.

Nonetheless, the Saudi Commission for Health Specialties (SCFHS) [12], which legally binds healthcare providers who practise medicine in Saudi Arabia to its requirements, has yet to update its policies to ensure that women have the right to make their own health decisions. Muaygil [13] discusses the issue of spousal consent, which is the requirement that Saudi women provide written consent from their spouse before they undergo any medical procedure that may affect their reproductive ability. Though spousal consent is not necessary from a legal or Islamic standpoint, the SCFHS still lists it as a requirement in its code of ethics. Muaygil [13] cites one case involving a married woman in her 50s who required an elective but medically indicated hysterectomy to alleviate her pelvic pain and bleeding. Although she was no longer able to bear children, her permission alone was not enough to authorise the procedure since she failed to provide spousal consent. Muaygil [13] argues that such policies cannot be ethically justified because they do not differentiate between therapeutic and contraceptive hysterectomies, violate women’s autonomy, are not supported by Islamic or legal considerations, and both physically and emotionally harm women.

However, Muaygil [13] differentiates between women of childbearing age and women who cannot bear children, arguing for spousal notification instead of spousal consent for the former population. Because hysterectomies prevent women from conceiving, she proposed this differentiation to protect women of childbearing age from domestic violence, requiring the spouse to be notified of the therapeutic need for the procedure. If the spouse were to attempt to stop the surgery, the physician would need to discuss its therapeutic benefits with him. If the spouse were to remain unconvinced, the hospital ethics service or, as a last resort, law services would need to intervene to prevent the woman from being harmed. Muaygil [13] also notes the SCFHS code’s contradiction in not also requiring a wife’s written consent, or even notification, when the husband undergoes a sterilisation procedure that produces the same outcome on men’s fertility as hysterectomies do for women. Thus, according to SCFHS policies, unlike Saudi women, who require spousal consent, Saudi men enjoy full autonomy in medical decisions without any regard for spousal consent, even though the circumstances and outcomes of their procedures are the same. Over the past decade, the Saudi government has enacted and changed many policies and regulations in favour of improving the social status of Saudi women. The SCFHS [12] should therefore also change its policies to support gender equity by respecting the autonomy of all adult patients within Saudi medical settings.^4^

In 2019, the Saudi Ministry of Health (MOH) [1] published guidelines that cover different aspects of informed consent in Saudi medical and research settings. These guidelines address medical conditions that cause infertility and childlessness and the required consent to permit appropriate interventions. In summary, the consent of a competent adult patient is sufficient when the patient is not married or if infertility is due to treatment and is only temporary regardless of marital status. In the event that the treatment will lead to permanent sterility but there is a clinical need for it, patient consent is sufficient for authorisation of the treatment even if the patient is married and regardless of whether they are male or female. In other words, while patients are asked to inform their husbands or wives of intervention as a form of spousal notification, they are not required to seek their consent.

Although it is promising that this approach has been adopted in national guidelines, the SCFHS must update its policies to be more consistent with MOH regulations if Saudi women’s autonomy is to be respected. Recall the case from the 2019 MOH guidelines [1, p. 15] where a woman’s spouse approved a cesarean on her behalf against her wishes. This seems inconsistent with respecting a patient’s autonomy and is thus incompatible with the intention of the Saudi guidelines for informed consent. If the condition of the patient and their foetus is stable, the patient’s refusal of the operation must be respected. In fact, her refusal of the operation could show that delegating decisional authority to her husband may not reflect her genuine preference if, for example, she was forced to undergo the operation due to socio-cultural influences that give her husband authority over her decisions. Instead, physicians should be obligated to check the patient’s genuine preferences concerning whether her husband is authorised to make decisions on her behalf. More broadly, all patients should be assessed to ensure that they are not being forced to give full authority to their families, since coercion undermines their autonomy. Given the cultural expectation that medical decisions should be made by male relatives, this is particularly important for female patients. If coercion is suspected, the patient’s decision should be discussed by the treating team so that they may devise practical steps to manage the issue.

A scenario where coercion is likely may involve a female patient who believes that she has no choice but to delegate decisions to her family and accept the authority of her male relatives, since they will ultimately insist on choosing what they believe is best for her. She may also fear upsetting her family, which could lead to domestic violence. Thus, socio-cultural influences that support the dominating role of families during management decisions can play a significant role in undermining patient autonomy, forcing females to accept the dictates of male relatives even if their autonomy to make medical decisions is protected by Saudi law and medical regulations. Although the presence of these regulations is not enough to ensure that patient autonomy will be respected in medical decisions, practical steps that may guarantee these practices can include updating the relevant regulations to adapt the preference approach in managing patients according to the model of shared decision-making and training physicians in respecting patient’s rights to make decisions in their own best interests. Family counselling could also take place with the support of social services to ensure that families understand the impact of their dominance over patient decisions. Elderly and illiterate parents are especially vulnerable in this regard, as their consent forms are usually filled out by their family members [7]. Thus, physicians must carefully assess elderly patients’ genuine preferences to ensure that their decisions are free of coercion or manipulation; however, this might not be easily assessed if their families are around them.

Families not only override patient autonomy when making management decisions or during the disclosure of diagnoses and prognoses, but also when patients who do not know their diagnoses and prognoses are enrolled in clinical research without their knowledge. The primary obstacle that research teams in Saudi Arabia encounter is in obtaining the consent of elderly and illiterate patients to participate in clinical trials, as these patients are typically surrounded by several family members. Researchers must first obtain the family’s permission in order to gain the patient’s consent [7]. This same issue also commonly prevents participation from Saudi women, as researchers may find it difficult to gain their consent without first obtaining the permission of their male guardians.

Clinical trials are important for fostering the knowledge required to improve medical practices. To gain approval to conduct clinical trials in Saudi medical settings, researchers must follow the executive regulations of Saudi law on the ethics of research on living creatures [15], which are issued by the National Committee for Bio-Medical Ethics (NCBE) [16].^5^ These regulations require the implementation of research procedures that are appropriate to the aims of the study, the protection of the informational privacy of patients in enrolment practices, and the provision of treatment to research participants who experience adverse events during a trial. The NCBE regulations [15] also obligate researchers to obtain patients’ informed consent before enrolling them in research, and state that the proposed research must maximise its benefits and minimise its harm for individuals and communities. However, these regulations do not acknowledge or account for family dominance over patient-participants’ decision-making during enrolment in clinical research, or address the issue of “therapeutic misconception” [2, p. 132], especially for patients who do not know about their diagnoses and prognoses due to familial control. When this occurs, patient-participants are unable to differentiate between clinical care and clinical research and are thus unable to protect their own interests [2]. Therefore, further regulations are necessary to ensure that patients cannot be enrolled in clinical trials unless they are adequately informed about their diagnoses and prognoses so they are in a position to provide valid consent for their participation. National guidelines and regulations for research ethics should provide adequate protections for research participants, particularly in relation to their exploitation or the prevalence of therapeutic misconceptions among those involved in clinical trials [7].

The Saudi guidelines for informed consent [1] specify two circumstances for organ donation: organ donation from a living person and a brain-dead person.^6^ The guidelines [1, p. 16] state that a health facility must adhere to the criteria for organ donation noted in the “the organ donation procedure guide issued by the Saudi Centre for Organ Transplantation.” When considering organ donations from a living person, health facilities must verify donors’ eligibility before initiating donation procedures and obtaining informed consent. Health facilities must also commit to treating complications resulting from living organ donation if they occur. When considering organ donations after brain death, health facilities and specialised medical staff are obligated to verify brain death according to the standards of the Saudi National Centre for Organ Transplantation [17]. Health facilities must also follow the informed consent criteria for organ donation that are included in the manual of organ donation procedures issued by the Saudi Centre for Organ Transplantation. In addition, health facilities must provide appropriate healthcare until the donation processes are completed, taking into account the dignity of the deceased and the psychological condition of his/her family [17].

Although many Muslims consider organ donation to be “an ongoing form of charity” insofar as it saves human lives and minimises suffering, [18, p. 12] family-centred decisions in organ donation frequently result in families refusing to honour the prior consent of brain-dead patients to donate their organs. This is an ethically problematic practice, which also occurs in a number of other jurisdictions.

The Holy Quran describes the virtuous act of saving a single human life as being comparable to saving all human lives:

[W]hoever kills a soul unless for a soul or for corruption [done] in the land—it is as if he had slain mankind entirely. And whoever saves one—it is as if he had saved mankind entirely [19].

According to a report from the Saudi Centre for Organ Transplantation, families refuse to allow organ donation from a deceased donor relative in 62% of cases [17]. This report does not specify whether these eligible donors consented to organ donation while they were alive. The rate of family refusal of donations is comparatively high. For example, in Cignarella et al.’s [20] retrospective study of medical records for potential organ donors who were admitted to the ICU of three Australian hospitals from 2012 to 2016, the authors found that 65% of patients’ families agreed to donate their relatives’ organs on their behalf. Among the remaining 35% who did not donate, 5% were due to patients who had previously refused to donate, while families declined donation in 11% of cases.

According to Al Sayyari et al. [21, p. 66], due to the extended family bonds in Saudi Arabia, a senior member with a high status in a family “may veto a decision for donation after death” even if the donor’s parents hold no objection to their child’s wishes to donate. This can also lengthen the consent process for organ donation after brain death. Reasons for family objection to donation can include the fear of torturing or disfiguring the donor’s body or the fear of delaying their interment, as Islam mandates immediate burial [21].

Darnell et al. [22] conducted a study that involved interviews with family members of potential donors at the time of their imminent death, aiming to explore the reasons behind family refusals of donation. They found that family refusal was primarily motivated by the decision to relieve their loved ones from the suffering they might experience during the process of organ donation, and to prevent the body of their loved ones from being cut or disfigured so that they may rest in peace. However, many justified their refusal on the basis of suggestions from their loved one that they did not want to be cut open to donate their organs. This is a common concern among many families of deceased donors from various cultural backgrounds. [22]

Cignarella et al. [20] remarks that current Australian policies in organ donation authorise families to override the prior consent of their relatives to donate organs. In response, the authors argue for the need to change Australian regulations so they remain consistent with international policies that ensure the wishes of patients who consent to organ donation will be respected despite any family objection, which the author of the current article believes is the right thing to do. In fact, the lack of policies in Saudi Arabia that are designed to protect against family dominance within medical contexts affects organ donations from both deceased relatives and living donors. As an example, Al Sayyari et al. [21, p. 70] explains that kidney donation from a living donor in Saudi Arabia may not always be a benevolent or uncoerced gesture. Extended family can often exert indirect pressure to convince a potential donor to donate a kidney, and this pressure can manifest in the form of “financial, psychological, familial, social or tribal” influences. This raises important ethical concerns. On occasion, a physician can only free the living donor of this coercion by making up “a medical excuse” so that the potential donor does not lose face in front of their family. As such, there is a particular need for policies to protect against family dominance in cases of live organ donation.

Islamic (shariah) law allows do not resuscitate orders (DNRs) for patients suffering from terminal illnesses. Under these orders, patients can receive all necessary treatment, hydration, nutrition, and pain control to die peacefully without receiving artificial resuscitation, as resuscitation to restore cardiac and/or respiratory function to arrested patients would only prolong their suffering in the absence of foreseeable benefits for prolonging their lives [23].

Such decisions should be made through discussions with competent patients who retain the right to refuse life-sustaining measures. However, Chamsi-Pasha and Albar [18] state that physicians in Islamic countries are reluctant to discuss DNRs with their competent patients, preferring instead to confer about the topic with patients’ families. When the condition of a patient who has not consented for a DNR becomes critical or they fall into a coma, the DNR decision is placed in the hands of at least three specialist physicians [24].

When the patient’s family refuse the DNR order which was issued by the patient themselves or by the medical team and the family requests efforts for resuscitation, they are allowed to do so by transferring the patient to another hospital where the patient could be resuscitated following cardiopulmonary arrest, as the signed DNR is only kept in the patient’s records and is not valid outside the hospital [23].

To prevent the prolongation of the patient’s suffering and prevent family dominance through demanding unnecessary resuscitation, the Saudi MOH should provide a clear policy to put an end to unnecessary resuscitation that is applicable in all healthcare settings [24]. Practice guidelines are also needed to prevent physicians from undermining patient autonomy when they abstain from discussing DNR decisions with their competent patients but do so with their families. In cases when a patient clearly shows a preference to know their options but their physician denies them the opportunity, it is possible that the patient may be resuscitated whether or not they want to be. When physicians discuss this important decision with family instead of the patient and without the patient’s authorisation, there are also ramifications to patient privacy and confidentiality [2]. Thus, to prevent family dominance that would harm the patient through prolongation of their suffering, the DNR decision should only be discussed with the family if the patient has delegated the decision for them to make or the patient has lost the competency to make decisions.

In light of the abovementioned considerations, physicians must avoid extremes when considering what to disclose to their patients or when seeking the consent of competent patients for health-related decisions. These extremes can involve enabling strong family paternalism by allowing families to prevent the disclosure of diagnoses and prognoses to patients or overriding patients’ consent for management options due to benevolent and protective cultural reasons. The other extreme is when physicians leave patients to make decisions without offering guidance or sharing in the decision-making process. Thus, the model of shared decision-making introduced in the following section, “Relational autonomy and individual autonomy in shared decision-making” section, will offer a path between these two extremes by checking patients’ preferences about medical disclosures and management options. Patients’ preferences for health-related decisions also need to be continually assessed since these may change with time or according to the seriousness of their medical circumstances. In addition to patients sharing in the decision-making process, physicians should also impart their medical expertise when recommending the best available course of action for their patients.

## Relational autonomy and individual autonomy in shared decision-making

This section presents a model of shared decision-making that accounts for both individual and relational concepts of autonomy, with the aim of minimising the potential for socio-cultural values to undermine patient autonomy.

Gómez-Vírseda et al. [25] argue that relational notions of autonomy that maintain a balance between individuality and relationality can promote patient wellbeing in a range of social contexts. They also claim that relational conceptions of autonomy are flexible enough to incorporate a range of socio-cultural values in relation to the family.

According to Gómez-Vírseda et al. [25], the process of decision-making in medical settings can take the form of the following four scenarios:

I. Compromised autonomy and paternalism: This occurs when a healthcare provider (medical paternalism) or the family (family paternalism) takes full control of the decision-making process and ignores or fails to check the preferences of a competent patient.
II. Delegating responsibilities: This occurs when the family and/or healthcare provider takes full control of the decision-making process because the patient prefers to let their caring and supportive family as well as the experienced physician take the lead.
III. Shared decision-making: In this scenario, there is more emphasis on individual autonomy and the relationship between the individual patient and the healthcare provider, which does not include the family unless the patient wants to include them. Here the emphasis is on identifying and respecting the patient’s values, preferences and beliefs, not just the disclosure of relevant clinical information. This process can also include elements of relational autonomy if the welfare of the patient is believed and decided (by the patient) to be inseparable from the welfare of his/her family.
IV. Prioritising individual autonomy: This occurs when patients make their own decisions without any contribution from the family or healthcare provider apart from what is required as part of the consent process.

Gómez-Vírseda et al. [25] hold that relational autonomy is relevant for the second and third decision-making scenarios mentioned above, wherein patients, their families and healthcare providers can all share in the decision-making process, or patients can delegate decision-making responsibilities to their families and/or healthcare providers. As will be discussed below, the relational approach can also open the door for discussions between secular-based and religion-based ethics in order to establish common ground in understanding respect for patient autonomy within familial and social contexts. Moreover, Gómez-Vírseda et al. [25] observe a gap in the literature regarding how to apply relational concepts of autonomy in clinical contexts, particularly during end-of-life care.

Various conceptions of relational autonomy^7^ have been proposed to serve as alternatives to the standard focus on individualism in maintaining self-governance over one’s own life. As an example, Dove et al. [26] views patients as relational beings who are necessarily dependent on those in their social surroundings, such as family and friends. They argue that applying a relational form of autonomy in medical practice and in research settings requires clinicians and researchers to respect patients’ and patient-participants’ need to involve their trusted family members and experienced healthcare providers in decision making. Clinicians and researchers can facilitate this by giving patients and patient-participants enough time to consent so they can consider their values and preferences within the context of their relationships. In allowing the patient’s family and those close to them a strong role in the decision-making process, relational conceptions of autonomy are more receptive to the dominant socio-cultural norms of Middle Eastern countries, where respect for extended family bonds is more prominent than the individualism and individual autonomy that is more common in Western countries.

From the perspective of the author’s interests in this article, respecting patients’ relational autonomy does not necessarily oppose the act of respecting their individual autonomy if both are incorporated into a model of shared decision-making that includes scenarios II and III from Gómez-Vírseda et al.’s [25] research. This approach enables patients to involve the people they trust when making management decisions while still providing them the space to choose or not to choose to involve their family. In this way, shared decision-making allows healthcare professionals to respect a patient’s values, beliefs and preferences, regardless of their family’s involvement in management decisions. This approach to shared decision-making avoids medical and family paternalism by only involving doctors and families in decisions when this is authorised by the patient themselves rather than doctors or families acting unilaterally. Thus, if a patient’s existing medical conditions are likely to benefit from delaying or limiting the disclosure of potentially distressing diagnoses and prognoses, doctors and families can in the author’s view justifiably fulfil weakly paternalistic roles on patients who are competent/decisionally capable. For example, truthful disclosures to patients with cardiac disease may significantly elevate stress levels to the point where their health deteriorates. In such cases, family involvement can attempt to ensure that management decisions that partially reflect the patient’s values and preferences are made in the best interests of the patient. The author of this article holds that this is acceptable only if the patient themselves authorises their family to do so.

This model of shared decision-making would be supported by continually checking the patient’s preferences for medical disclosures and management decisions to clarify whether they are more in line with individual or relational understandings of autonomy. In this way, patients are able to provide clear guidance to healthcare professionals for when it is appropriate to contact people they trust, and to authorise them to make the best decisions on their behalf when their condition requires it.^8^

Dove et al. [26] support this kind of shared decision-making model by arguing that relational approaches are acceptable only if the patient’s voice has not been replaced by another’s. As discussed in previous sections, vulnerable patients can suffer from strongly paternalistic practices that override individual or relational patient autonomy (e.g., when the consent of others, such as the spouse, is mandatory before the authorisation of certain medical procedures or enrolment in research). Thus, for shared decision-making to genuinely reflect relational forms of autonomy, it must maintain safeguards against medical and family paternalism by ensuring that the relational component of patient consent remains uncoerced by any means, such as when competent/decisionally capable patients are not fortunate enough to have a caring spouse and/or family member who can be trusted to make management decisions in their best interests. Therefore, in order to eliminate concerns of external coercion, which negatively impact both relational and individual models of autonomy, the stated preferences of patients who are at higher risk of being in abusive and controlling relationships should be carefully assessed. In cases where a patient is in conflict with their family, the patient should have the final say in the decision-making process. Moreover, adopting a relational form of autonomy can contribute to better protections of confidentiality by encouraging patients to share their private information with their families during decision-making processes, at least when they prefer to do so, which is less concerning under a relational model than it is for individual autonomy.

The suggested preference approach involves the adoption of a model of shared decision-making that protects patients’ preferences from being overridden by both family paternalism and medical paternalism. This approach involves checking patients’ preferences for decision-making to ensure that patients who prefer to maintain control over their medical decisions are not disenfranchised by their families, regardless of their gender, age or socio-cultural background. The preference approach should be more detailed and autonomy-focused than what is already practised in Saudi medical settings, where patient consent is obtained but their preferences for the disclosure and the extent of family involvement in decisions are not adequately checked.

The author recommends that in every medical encounter, all patients’ preferences for knowing about their diagnoses and prognoses in addition to the roles (if any) they would like their families to play in their management decisions should be routinely checked.

Care should be taken when checking elderly and female patients’ preferences for medical decisions, as they may want to be informed of their diagnoses and prognoses personally, but may fear disappointing or angering their family for this decision. In such cases, their preferences for shared or delegated decisions to their family need to be verified without their family’s presence to ensure they are not being coerced or manipulated (i.e., to confirm that their preferences are genuinely autonomous).

Nonetheless, even if patients decide to keep their family out of the decision-making process, there are beneficial reasons for them to choose otherwise. For example, because disclosing bad diagnoses and prognoses to cancer patients is typically stressful, it is possible that such disclosures could play a major role in worsening patients’ physical and emotional conditions. Consider a patient with a medical history of cardiovascular disease (CVD),^9^ depression or anxiety or any medical condition that may be worsened by the stress of a full disclosure of a cancer diagnosis. In a case like this, the patient would need to be assessed according to reliable criteria for co-existing conditions. Any such assessment needs to be accurate enough to justify delaying or withholding information. For example, the Framingham Heart Study estimates a patient’s risk of experiencing worsening CVD,^10^ while the risk of suicide is commonly assessed in patients with histories of major depression and anxiety disorders.^11^ Although such instruments do not specify stress as one of their criteria of assessment, they still provide an indication of a person’s current mental and emotional state and risk for either a cardiac event or suicide. Therefore, these measures are helpful in predicting the effects of stress on disclosures of bad diagnoses and prognoses.

The preference approach must be grounded in a biopsychosocial approach to caring for patients in Saudi medical settings. This involves providing a comprehensive assessment of a patient’s psychological and social wellbeing in addition to their physical wellbeing, since all these domains contribute to the patients’ overall welfare.

Under this approach, the team directly responsible for the patient’s management is required to assess the patient’s capacity to handle the disclosure of bad news and to obtain informed consent for subsequent medical interventions. Other healthcare service providers, such as social workers, oncologists and the other medical specialists the patient needs to treat their medical condition, should also play a role in supporting the patient’s physical, psychological and social wellbeing. This support could help prepare the patient to accept and understand their diagnosis and prognosis, which is essential in providing care to a patient during a difficult diagnostic and therapeutic process, a factor which is all too often neglected.

The extent of the family’s contributions to the decision-making process should be determined by competent patients. This means that whether the family shares in the patient’s health decisions, takes full responsibility for management decisions, or are kept out of the process entirely, should depend on the patient’s preferences. Patients with co-existing medical diagnoses who give authority to their family to make medical decisions on their behalf must understand that the family may insist on delaying or limiting medical information from being disclosed to them if their medical condition justifies the family’s decision to do so. However, even if a patient prefers to keep their family out of the decision-making process, physicians can justifiably delay or limit the disclosure of medical information to prevent significant harm to a patient if that patient is not imminently dying but has pre-existing conditions that can be worsened by full disclosures.

## Conclusion

In this article, the author identified a range of harms that arise in Saudi medical settings due to the influence of cultural values that support a role for families in medical decision-making that often overrides patient autonomy. Whether it involves medical disclosures, protecting patient privacy and confidentiality or consent, the priority given to family decision-making leaves little (if any) room for a genuine consideration of patient preferences, especially if the patients are female or elderly. This is reflected in the lack of national policies that are specifically intended to protect patient autonomy from family dominance over decisions in relation to medical disclosures, confidentiality and consent. In the absence of such policies, healthcare providers too often acquiesce to the cultural norm of family dominance in medical decision-making. Against this background, the author argued for the implementation of a model of shared decision-making that accounts for both individual and relational conceptions of autonomy in placing an obligation on physicians to maintain safeguards against strong medical and family paternalism.

The continual assessment of patient preferences for medical disclosures and consent should be used in all medical encounters with patients in Saudi medical settings to verify the extent to which patients’ families are authorised to participate in medical decision-making. Physicians have an obligation to ensure that their patients fully understand that while medical reasons to authorise the family to make decisions on their behalf are acceptable, socio-cultural expectations are not. The author also argued that if a patient has pre-existing conditions that could worsen from full disclosures, risk assessments of their existing condition may justify physicians delaying or limiting the disclosure of medical information to avoid exacerbating their condition.

## Data Availability

Not applicable.

## Abbreviations

MOH: The Saudi Ministry of Health
CS: Caesarean section
SCFHS: The Saudi Commission for Health Specialties
NCBE: The National Committee for Bio-Medical Ethics
ICU: Intensive care unit
DNRs: Do not resuscitate orders
UK: United Kingdom
CVD: Cardiovascular disease

## Footnotes

1 It may be ethically justifiable under certain circumstances (e.g., to save a patient’s life).

2 Altoaimy[11, p. 1] followed “a corpus-assisted discourse studies approach” by collecting and analysing Twitter debates in Arabic discussing the ban on female driving in Saudi Arabia in the last three months of 2015.

3 The Twitter account of the Saudi Passports Department announced in Arabic that “In implementation of the noble Royal Decree No. (M/134) dated 11/27/1440 AH, passports began receiving requests from women aged 21 years and over to issue or renew passports, and to travel outside the kingdom without the need for a permit” [14].

4 The code of ethics for healthcare practitioners published by the SCFHS in 2014 [12]. The author could not find any recent updates for this guideline.

5 The NCBE’s interests include bio/medical research ethics and their related applications in hospitals, universities and research institutes. The committee operates under the supervision and authority of King Abdulaziz City for Science and Technology (KACST), which chairs the committee [16].

6 The guidelines do not address donations after cardiac death.

7 There is a range of available conceptions on relational autonomy, which are commonly criticised for being vague (see, for example, Gómez-Vírseda et al. [25]).

8 Similar to nominating a surrogate decision-maker, where decisions are made on behalf of those who are incompetent, this process involves advanced directives and delegations for someone to make decisions on behalf of competent patients who may temporarily become incompetent (due to a high risk of cardiac or psychiatric events). Thus, this practice functions as a safeguard against family or medical paternalism.

9 Emotional stress has been reported to trigger acute cardiac events in patients with other risk factors for CVD, primarily by causing “disturbances of inflammatory, haemostatic, and autonomic processes are likely to be the mechanisms by which short-term psychological stress triggers acute myocardial infarction.” Moreover, stress management, among other lifestyle modifications, has been found to be beneficial in protecting cardiovascular health [27, p. 361].

10 For estimations on the risk of recurring CVD events: [28]. For estimations on the risks of new CVD events: [29].

11 Scale for estimating the risk of suicide: [30].
